# Giant Hepatobiliary Cystadenoma Requiring Biliary Reconstruction in a Cat: A Case Report

**DOI:** 10.1002/vms3.71174

**Published:** 2026-08-14

**Authors:** Abdolrazagh Rostami, Milad Samadipoor, Pouran Mohammadi, Solmaz Chegeni, Arman Abdous

**Affiliations:** ^1^ Private Practice Tehran Iran; ^2^ Department of Clinical Sciences Faculty of Veterinary Medicine S.R.C., Islamic Azad University Tehran Iran; ^3^ Iran University of Medical Sciences Tehran Iran; ^4^ Faculty of Veterinary Medicine Ka.C., Islamic Azad University Karaj Iran

**Keywords:** biliary reconstruction, case report, cat, computed tomography, hepatobiliary cystadenoma

## Abstract

**Background:**

Hepatobiliary cystadenoma is an uncommon benign biliary tumour in domestic cats (*Felis catus*) that may mimic cystadenocarcinoma. Although imaging provides important diagnostic information, histopathological examination is required for confirmation. This case was notable for the exceptionally large lesion and the need for biliary reconstruction following surgical excision.

**Case Presentation:**

A 7‐year‐old neutered male domestic shorthair cat weighing 8 kg was presented with several weeks of vomiting, inappetence and abdominal distension. Serum biochemistry demonstrated a cholestatic pattern with increased alkaline phosphatase and gamma‐glutamyl transferase activities. Abdominal radiographs revealed a cranial abdominal mass, and ultrasonography identified a multiloculated cyst with thin septa and no mural nodules. Contrast‐enhanced computed tomography confirmed a 163 × 148 × 136 mm cystic hepatic mass with homogeneous fluid attenuation, non‐enhancing septa and no solid components. Exploratory laparotomy enabled complete excision with preservation of hepatic tissue. Partial resection of the common bile duct followed by end‐to‐end anastomosis was performed to restore bile flow. Histopathological examination confirmed hepatobiliary cystadenoma. Recovery was uncomplicated, and at the 3‐month follow‐up the cat was clinically normal, with no ultrasonographic evidence of recurrence.

**Conclusions:**

Large multiloculated hepatic cystic lesions with homogeneous fluid contents and no enhancing mural nodules should prompt consideration of hepatobiliary cystadenoma in cats. Complete surgical excision with preservation or reconstruction of biliary structures may result in a favourable short‐term outcome. Longer clinical, biochemical, and imaging follow‐up is recommended.

## Graphical abstract

A seven‐year‐old cat had a 16.3 cm multiloculated hepatobiliary cystadenoma occupying most of the abdomen. Complete excision required partial common bile duct resection followed by tension‐free end‐to‐end anastomosis. Recovery was uncomplicated, and no recurrence or clinically evident anastomosis‐related complication was detected at the 3‐month follow‐up.

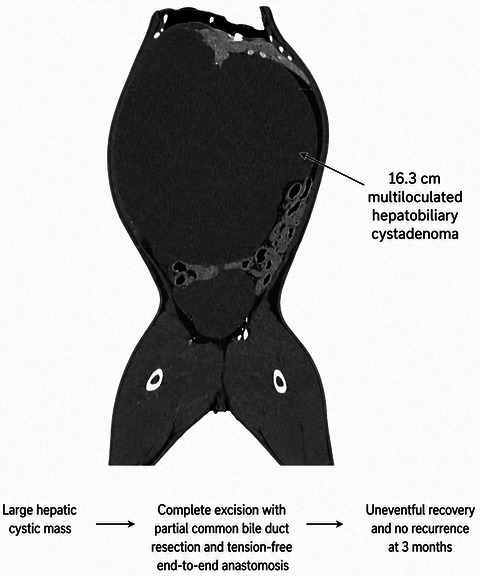

**Abbreviations**
ALP: alkaline phosphatase
ALT: alanine aminotransferase
AST: aspartate aminotransferase
CT: computed tomography
GGT: gamma‐glutamyl transferase
H&E: haematoxylin and eosin
HU: Hounsfield unit

## 1 Background

Hepatobiliary cystadenoma is an uncommon benign cystic neoplasm of the biliary epithelium that has been sporadically reported in domestic cats (*Felis catus*) (Adler and Wilson 1995; Tiwananthagorn et al. 2018; Elpiner 2020). Its true incidence remains uncertain, but it is considered an uncommon cause of hepatic cystic disease compared with congenital simple cysts, abscesses or malignant tumours (Tiwananthagorn et al. 2018; Griffin 2019).

Historically, published descriptions have largely consisted of individual reports and small case series (Peterson 1984; Adler and Wilson 1995; Trout et al. 1995; Nyland et al. 1999; Vala et al. 2009; Tiwananthagorn et al. 2018). Reported cysts have ranged from microscopic lesions to masses measuring several centimetres, and masses up to 12.5 cm have been described (Aiello et al. 2016). Complete surgical excision has been the standard therapeutic approach when feasible (Trout et al. 1995; Trout 1997).

The present case was notable for a lesion measuring 16.3 cm in its greatest dimension and for the requirement of common bile duct resection and reconstruction. To the authors’ knowledge, direct common bile duct reconstruction during the excision of a feline hepatobiliary cystadenoma has not previously been reported. This report details the diagnostic imaging, surgical approach, histopathological findings, and short‐term outcome.

## 2 Case Presentation

A 7‐year‐old neutered male domestic shorthair cat weighing 8 kg was presented with several weeks of intermittent vomiting, inappetence and progressive abdominal distension. The owner had sought multiple veterinary consultations before referral. On admission, the cat was bright and alert with unremarkable vital parameters. Abdominal palpation revealed a firm cranial abdominal mass without apparent pain. No clinical icterus was observed.

A complete blood count and serum biochemistry panel were performed. The profile was notable for a cholestatic pattern, with increased alkaline phosphatase (ALP) and gamma‐glutamyl transferase (GGT) activities. Alanine aminotransferase (ALT) activity was moderately increased, while aspartate aminotransferase (AST) activity was slightly above the reference interval. These findings were consistent with hepatobiliary dysfunction (Adler and Wilson 1995; Tiwananthagorn et al. 2018). Serial results are summarised in Table 1.

**TABLE 1:** Haematology and serum biochemistry results.

Parameter	Preoperative	Postoperative Day 1	Postoperative Day 3	3‐month follow‐up	Reference interval
PCV (%)	35	33	34	37	30–45
WBC (×10^9^/L)	10.2	11.5	9.8	8.7	5.5–19.5
Platelets (×10^9^/L)	350	320	310	340	300–800
ALT (U/L)	145	130	98	62	20–100
AST (U/L)	65	59	52	48	15–60
ALP (U/L)	225	210	180	95	10–90
GGT (U/L)	18	15	12	6	0–10
Total bilirubin (µmol/L)	12	10	8	5	0–10
Albumin (g/L)	29	28	30	32	28–40
BUN (mmol/L)	7.0	6.8	6.5	7.1	5.4–12.0
Creatinine (µmol/L)	130	125	120	128	70–165
Sodium (mmol/L)	150	148	149	151	147–156
Potassium (mmol/L)	4.1	4.3	4.2	4.0	3.5–5.1
Chloride (mmol/L)	115	113	114	116	112–129

*Note*: Reference intervals were provided by the laboratory that performed the analyses.

Abbreviations: ALP, alkaline phosphatase; ALT, alanine aminotransferase; AST, aspartate aminotransferase; BUN, blood urea nitrogen; GGT, gamma‐glutamyl transferase; PCV, packed cell volume; WBC, white blood cell count.

Survey abdominal radiography was initially performed as a screening examination using right lateral and ventrodorsal projections. These two orthogonal views demonstrated a markedly enlarged soft‐tissue opacity occupying the cranial abdomen, displacing adjacent viscera and producing diffusely reduced abdominal serosal detail (Figure 1A,B). Because the large space‐occupying lesion was clearly identified and the owner subsequently elected further diagnostic evaluation, abdominal ultrasonography and contrast‐enhanced computed tomography (CT) were prioritised over an additional left lateral radiographic projection. These modalities were expected to provide more clinically useful information regarding the lesion's origin, internal architecture, anatomical relationships and suitability for surgical excision.

**FIGURE 1:**
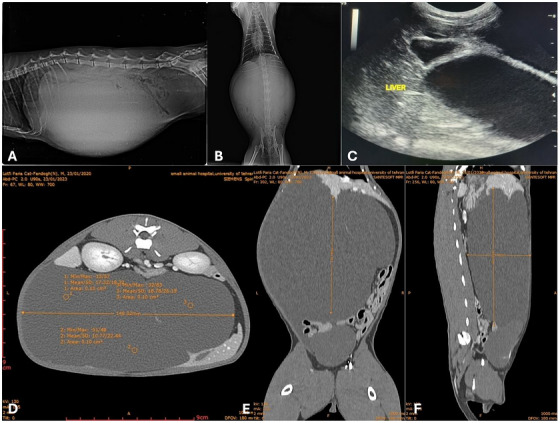
Diagnostic imaging findings of the hepatobiliary cystadenoma. (A) Right lateral abdominal radiograph showing a large, space‐occupying, soft‐tissue opacity in the cranial abdomen, with caudal displacement of the intestinal loops and reduced abdominal serosal detail. (B) Ventrodorsal abdominal radiograph showing the large soft‐tissue opacity occupying most of the cranial and mid‐abdomen. The “L” marker indicates the cat's left side. (C) Sagittal abdominal ultrasonographic image showing an anechoic, multiloculated cystic lesion associated with the liver and divided by thin, hyperechoic septa. No mural nodule or irregular wall thickening is visible. (D) Transverse portal venous‐phase, contrast‐enhanced computed tomographic image showing homogeneous fluid attenuation, thin non‐enhancing septa. (E) Dorsal reconstruction demonstrating the extent of the lesion and its association with the right medial hepatic lobe. (F) Sagittal reconstruction showing the craniocaudal extent of the mass and displacement of adjacent abdominal organs. The lesion measured 163 × 148 × 136 mm and contained no enhancing solid components.

Abdominal ultrasonography was performed using a SonoMaxx 100 hand‐held veterinary ultrasound system (BMV Technology) equipped with a microconvex probe with a frequency range of 5.0–8.0 MHz. The examination revealed a large cystic mass arising from the right medial hepatic lobe, with a thin wall and anechoic contents subdivided by fine hyperechoic septa into three communicating compartments. No mural nodules or irregular wall thickening were observed (Figure 1C) (Elpiner 2020; Griffin 2019).

Contrast‐enhanced abdominal CT was performed using a 16‐slice multidetector CT scanner (SOMATOM go.Now, Siemens Healthineers, Forchheim, Germany). Acquisition parameters were as follows: slice thickness, 0.6 mm; pitch, 0.8; tube voltage, 120 kVp; tube current, 150 mAs; and matrix size, 512 × 512. Non‐contrast scans were followed by intravenous administration of iohexol (300 mg iodine/mL; Omnipaque 300, GE Healthcare, Chicago, IL, USA) at a dose of 600 mg iodine/kg body weight through a cephalic catheter. Post‐contrast scans were acquired at 30 s (arterial phase), 60 s (portal venous phase), and 180 s (delayed phase). Multiplanar reconstructions in transverse, dorsal, and sagittal planes were generated for comprehensive assessment.

CT confirmed a multiloculated cystic lesion arising from the right medial hepatic lobe. Lesion dimensions were obtained using the vendor‐provided measurement application on the CT workstation. The images were independently reviewed and measured by three board‐certified veterinarians in diagnostic imaging, and the reported dimensions of 163 × 148 × 136 mm represented the mean of their measurements. The mass was encapsulated by a thin, well‐defined rim and contained homogeneous fluid with attenuation values of 16–20 Hounsfield units, measured at multiple sites. Internal non‐enhancing septa were evident, and no contrast‐enhancing solid components were identified. The lesion occupied approximately 60%–70% of the abdominal cavity and displaced adjacent abdominal organs peripherally. Multiplanar reconstructions confirmed its origin within the right medial hepatic lobe (Figure 1D–F). Post‐contrast CT can assist with the localisation of feline hepatic masses when substantial displacement of adjacent structures is present (Bentley et al. 2024).

Given the size and imaging characteristics, the principal differential diagnoses included hepatobiliary cystadenoma, biliary cystadenocarcinoma, a simple hepatic cyst, an intrahepatic abscess and an organised haematoma (Table 2) (Griffin 2019). The absence of mural nodules or contrast‐enhancing soft‐tissue elements favoured a benign cystic lesion, although definitive diagnosis required histopathological examination.

**TABLE 2:** Differential diagnoses of hepatic cystic lesions in cats.

Diagnosis	Imaging features	Cytological or histological features	Clinical context
Hepatobiliary cystadenoma	Large multiloculated cyst, thin septa, homogeneous fluid attenuation, no mural nodules and non‐enhancing contents	Cuboidal or columnar biliary epithelium, fibrous stroma and no atypia	Middle‐aged to older cats; nonspecific signs such as vomiting, inappetence and abdominal distension
Biliary cystadenocarcinoma	Multiloculated cyst with mural nodules, irregular wall thickening, enhancing soft‐tissue elements or possible biliary obstruction	Cellular atypia, mitotic activity and stromal invasion	Aggressive behaviour; may cause icterus or ascites
Simple hepatic cyst	Unilocular and thin‐walled, anechoic on ultrasonography, fluid attenuation on CT, no septa or nodules	Flattened epithelial lining without atypia	Often incidental and usually asymptomatic
Hepatic abscess	Complex cyst with echogenic debris, gas echoes, irregular wall or rim enhancement	Suppurative inflammation, with or without identifiable bacteria	Fever, leucocytosis, abdominal pain or systemic illness
Haematoma	Mixed echogenicity, fluid‐fluid levels and variable attenuation depending on stage	Blood and blood‐degradation products without an epithelial lining	History of trauma or coagulopathy

Abbreviation: CT, computed tomography.

An exploratory laparotomy was performed through a ventral midline celiotomy extending from the xiphoid to the pubis. Exploration confirmed a large, multiloculated cystic mass originating from the right medial hepatic lobe, extending towards the gallbladder fossa and closely associated with the common bile duct. To facilitate manipulation, the cyst was decompressed by aspiration of 480 mL of serous fluid through a purse‐string suture placed in the cyst wall. The cyst was then dissected from the surrounding hepatic parenchyma using a combination of blunt dissection with Metzenbaum scissors and a vessel‐sealing device (LigaSure, Covidien Medtronic) to achieve haemostasis while minimising thermal injury to the adjacent liver tissue (Johnston and Tobias 2018). The dissection plane was identified by following the fibrous capsule of the cyst, which separated readily from the normal hepatic parenchyma except in the region of adherence to the common bile duct. Care was taken to preserve the right medial hepatic lobe parenchyma.

Owing to firm adhesions between the cyst wall and the common bile duct, partial resection of the duct was required. Biliary continuity was restored by end‐to‐end common bile duct anastomosis using 5–0 polydioxanone (PDS, Ethicon) suture in a simple interrupted pattern (Jifcovici et al. 2021). A leak test was performed by infusing sterile saline through a catheter inserted into the proximal common bile duct while occluding the distal segment. No leakage was observed. A pedicled omental graft was placed around the anastomosis to enhance vascularisation and reduce the risk of bile leakage or stricture formation. End‐to‐end anastomosis was chosen over choledochoduodenostomy because sufficient ductal length was available for tension‐free apposition, thereby preserving normal biliary anatomy and avoiding the risk of ascending cholangitis (Hayes et al. 2019). The abdomen was lavaged with warm sterile saline, and haemostasis was confirmed before routine closure (Figure 2). The procedure was completed without intraoperative complications, and the cat remained haemodynamically stable throughout.

**FIGURE 2:**
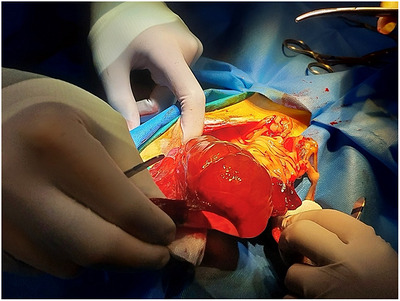
Intraoperative photograph obtained during exploratory laparotomy showing the exposed, multiloculated cystic mass during mobilisation from the right medial hepatic lobe. Moistened laparotomy sponges were used to isolate the operative field and protect adjacent tissues.

Postoperative management was initiated immediately following recovery from anaesthesia. Multimodal analgesia consisted of methadone hydrochloride (Comfortan, Dechra Veterinary Products Inc.; 0.2 mg/kg) administered intravenously every 6 h for the first 24 h, followed by buprenorphine hydrochloride (Vetergesic, Ceva Animal Health; 0.02 mg/kg) administered by the buccal route every 8 h for an additional 72 h. Pain was assessed every 4 h using the Feline Grimace Scale, with a score of 4/10 or greater prompting clinical reassessment and rescue analgesia (Evangelista et al. 2019).

Perioperative antimicrobial therapy consisted of cefazolin at 22 mg/kg administered intravenously every 8 h for 48 h. This was followed by oral amoxicillin‐clavulanate potassium (Clavamox, Zoetis Inc.; 62.5 mg per cat) every 12 h to complete a total treatment period of 7 days.

Nutritional support was provided by assisted feeding with a high‐protein recovery diet (Royal Canin Recovery Feline, Royal Canin) beginning 12 h after surgery, with daily caloric intake calculated at 60 kcal/kg and divided into four small meals. Postoperative monitoring included assessment of heart rate, respiratory rate, body temperature, pain score and incisional appearance every 4 h for the first 48 h and every 8 h thereafter. Intravenous crystalloid fluids (Hartmann's solution, Vetivex 11, Dechra Regulatory B.V.) were administered at 3 mL/kg/h for 24 h. Serum biochemistry was repeated at 24 and 72 h postoperatively to monitor hepatic function and cholestatic parameters (Table 1). The cat's appetite, activity level, and body weight were recorded daily.

By postoperative Day 3, serum biochemistry demonstrated continued improvement in the cholestatic parameters, with ALP activity decreasing to 180 U/L and GGT activity decreasing to 12 U/L. Haematological values remained stable (Table 1), and the cat was eating independently, moving comfortably and showing no signs of pain or surgical‐site complications. Recovery was uncomplicated, with progressive improvement in appetite and activity from postoperative Day 3 onwards.

Grossly, the excised specimen was a large, well‐circumscribed, multiloculated cyst filled with low‐protein, acellular fluid. Tissues fixed in 10% neutral buffered formalin were processed and stained with haematoxylin and eosin (H&E). Histologically, the cystic spaces were lined by a single layer of cuboidal to columnar biliary‐type epithelium with round vesicular nuclei and granular chromatin, supported by fibrous collagenous stroma. The epithelial cells had abundant cytoplasm and uniform nuclei. Mitotic figures were not observed, and there was no evidence of invasion or malignancy. These findings confirmed the diagnosis of hepatobiliary cystadenoma (Figure 3) (Adler and Wilson 1995; Nyland et al. 1999).

**FIGURE 3:**
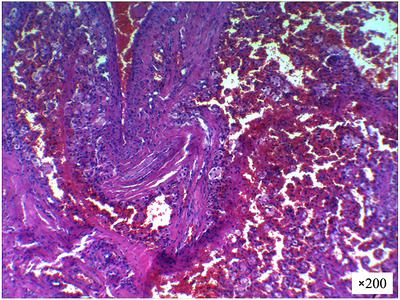
Histopathological appearance of the resected hepatobiliary cystadenoma. The cystic spaces are lined by cuboidal to low columnar biliary‐type epithelial cells supported by fibrovascular and collagenous stroma. Cellular atypia, mitotic activity, and stromal invasion are not identified. Haematoxylin and eosin stain; original magnification ×200.

Follow‐up examinations showed continued clinical improvement without reported complications. The cat returned to normal activity and appetite. At the 3‐month follow‐up, serum biochemistry showed that ALP activity had decreased to 95 U/L and GGT activity was within the reference interval (Table 1). Abdominal ultrasonography showed no evidence of recurrence.

## 3 Discussion

The imaging characteristics observed in this case aligned with published descriptions of benign biliary cystic lesions in cats. Mural nodularity, irregular wall thickening or contrast‐enhancing solid components may raise concern for cystadenocarcinoma, whereas uniform fluid attenuation and non‐enhancing septa are more consistent with cystadenoma (Adler and Wilson 1995; Tiwananthagorn et al. 2018; Griffin 2019). Nevertheless, cross‐sectional imaging cannot provide absolute diagnostic certainty and histopathological examination remains necessary.

The present case differed from the previously cited reports in two important respects. First, the lesion dimensions substantially exceeded those documented in the earlier literature. Peterson (1984) reported an intrahepatic biliary cystadenoma in a cat. Adler and Wilson (1995) described 13 cases with cysts ranging from microscopic lesions to several centimetres. Trout et al. (1995) reported five surgically treated cases, and Nyland et al. (1999) described three cases with cyst diameters of 2–6 cm. Tiwananthagorn et al. (2018) documented multifocal cysts measuring 0.5–4.0 cm and Vala et al. (2009) reported an additional case in a geriatric cat. The Merck Veterinary Manual describes biliary cystadenomas ranging from 1 mm to 8 cm and masses up to 12.5 cm (Aiello et al. 2016). The 16.3 cm lesion in the present cat was therefore exceptionally large.

Second, the previously cited surgically managed cases were treated by cyst excision or partial hepatectomy without common bile duct reconstruction. In the present case, firm adherence of the cyst wall to the common bile duct necessitated partial ductal resection and reconstruction. This surgical approach has not been previously documented in the literature concerning feline hepatobiliary cystadenoma.

Surgical planning should balance complete lesion removal with maximal preservation of functional hepatic and biliary anatomy. Enucleation may be appropriate for well‐circumscribed cystic lesions that are separable from the surrounding parenchyma, whereas partial hepatectomy may be required when a lesion involves or distorts a defined lobe or when a dissection plane is unclear (Johnston and Tobias 2018). In the present case, meticulous dissection and excision of the cyst from the right medial hepatic lobe were feasible. However, close adherence to the common bile duct required partial resection and end‐to‐end ductal anastomosis to maintain physiological bile flow. Key surgical principles included gentle tissue handling, secure biliary suturing with fine monofilament, leak testing before closure and omental support. Choledochoduodenostomy is an alternative when direct anastomosis is not possible but was not required in this case (Hayes et al. 2019).

Histopathological examination remains the definitive diagnostic step for biliary cystic lesions. H&E sections demonstrating a single layer of cuboidal to columnar biliary‐type epithelium over fibrous stroma, without atypia, mitotic activity or invasion, are consistent with cystadenoma (Adler and Wilson 1995; Nyland et al. 1999). Immunohistochemical testing was not performed in the present case because the routine histological findings were considered diagnostic.

The prognosis after complete excision of hepatobiliary cystadenoma in cats is generally favourable when biliary drainage is preserved (Nyland et al. 1999; Trout 1997). Complete excision without ductal resection therefore remains preferable when feasible. Biliary reconstruction introduces potential complications, including anastomotic leakage, stricture formation and ascending cholangitis. Published feline evidence remains limited, although successful end‐to‐end common bile duct anastomosis has been reported (Jifcovici et al. 2021). In the present case, no clinically evident anastomosis‐related leakage, stricture or cholangitis was detected during the 3‐month follow‐up period. However, the limited follow‐up duration precludes conclusions regarding long‐term safety. Continued clinical examination, serum biochemistry and abdominal ultrasonography are therefore recommended.

## 4 Conclusions

In conclusion, a very large, multiloculated hepatic cyst with homogeneous fluid attenuation and no mural nodules or contrast‐enhancing soft‐tissue elements should prompt consideration of hepatobiliary cystadenoma in cats. Clinicians should combine cross‐sectional imaging with definitive histopathological examination, plan for complete excision while preserving or reconstructing biliary outflow as needed, and implement long‐term clinical, laboratory and imaging follow‐up. The favourable 3‐month outcome in this cat is encouraging, but longer follow‐up is required to assess recurrence and anastomotic complications.

## Author Contributions

**Abdolrazagh Rostami**: conceptualisation, formal analysis, investigation, methodology, writing – original draft, writing – review and editing. **Arman Abdous**: conceptualisation, methodology, supervision, writing – original draft, writing – review and editing. **Milad Samadipoor**: data curation, investigation, writing – review and editing. **Pouran Mohammadi**: data curation, investigation, writing – review and editing. **Solmaz Chegeni**: data curation, formal analysis, writing – review and editing.

## Funding

The authors have nothing to report.

## Ethics Statement

The authors confirm that the ethical policies of *Veterinary Medicine and Science*, as outlined in the journal's author guidelines, have been adhered to. The case was reviewed and approved by the Ethics Review Committee of the Tehran Pet Veterinary Hospital, Tehran, Iran (approval number VH‐ERC‐2025‐041). Written informed consent was obtained from the cat's owner for all diagnostic, surgical and therapeutic procedures performed.

## Consent

Written informed consent was obtained from the cat's owner for publication of the clinical information and diagnostic, surgical, and histopathological images included in this case report. A copy of the consent documentation is available for review by the Editor upon request.

## Conflicts of Interest

The authors declare no conflicts of interest.

## Data Availability

The de‐identified data supporting the findings of this case report are included in the article. Additional de‐identified information is available from the corresponding author upon reasonable request, subject to the protection of owner and patient confidentiality.
